# Production and Increase of Secretion of Milk by the Ricinus Communis

**Published:** 1880-05

**Authors:** 


### PRODUCTION AND INCREASE OF SECRETION OF MILK BY THE RICINUS COMMUNIS.

MM. Boncher and Foussagivis have established the efficaciousness of this method of increasing or re-establishing the secretion of milk. It is used thus: a handful of the leaves of the ricinus communis are boiled in a litre of water. The breasts are bathed with the decoction for 15 or 20 minutes; there is then applied to the nipples a poultice made with a part of the same leaves, and they are left on till they become dry. The result is obtained after a few hours; but if the secretion is very tardy, we may add to this the employment of fumigation of the boiled leaves directed to the genital organs.—*Medical Press and Circular.*

